# Method to simulate distal flow resistance in coronary arteries in 3D printed patient specific coronary models

**DOI:** 10.1186/s41205-020-00072-7

**Published:** 2020-08-06

**Authors:** Kelsey N. Sommer, Vijay Iyer, Kanako Kunishima Kumamaru, Ryan A. Rava, Ciprian N. Ionita

**Affiliations:** 1 Department of Biomedical Engineering, University at Buffalo, Buffalo, NY USA; 2 Canon Stroke and Vascular Research Center, University at Buffalo, Buffalo, NY USA; 3 University at Buffalo Cardiology, University at Buffalo Jacobs School of Medicine, Buffalo, NY USA; 4 Juntendo University Department of Radiology, Tokyo, Japan

**Keywords:** 3D printing, Coronary artery disease, Coronary CTA, Flow resistance

## Abstract

**Background:**

Three-dimensional printing (3DP) offers a unique opportunity to build flexible vascular patient-specific coronary models for device testing, treatment planning, and physiological simulations. By optimizing the 3DP design to replicate the geometrical and mechanical properties of healthy and diseased arteries, we may improve the relevance of using such models to simulate the hemodynamics of coronary disease. We developed a method to build 3DP patient specific coronary phantoms, which maintain a significant part of the coronary tree, while preserving geometrical accuracy of the atherosclerotic plaques and allows for an adjustable hydraulic resistance.

**Methods:**

Coronary computed tomography angiography (CCTA) data was used within Vitrea (Vital Images, Minnetonka, MN) cardiac analysis application for automatic segmentation of the aortic root, Left Anterior Descending (LAD), Left Circumflex (LCX), Right Coronary Artery (RCA), and calcifications. Stereolithographic (STL) files of the vasculature and calcium were imported into Autodesk Meshmixer for 3D model optimization. A base with three chambers was built and interfaced with the phantom to allow fluid collection and independent distal resistance adjustment of the RCA, LAD and LCX and branching arteries. For the 3DP we used Agilus for the arterial wall, VeroClear for the base and a Vero blend for the calcifications, respectively. Each chamber outlet allowed interface with catheters of varying lengths and diameters for simulation of hydraulic resistance of both normal and hyperemic coronary flow conditions. To demonstrate the manufacturing approach appropriateness, models were tested in flow experiments.

**Results:**

Models were used successfully in flow experiments to simulate normal and hyperemic flow conditions. The inherent mean resistance of the chamber for the LAD, LCX, and RCA, were 1671, 1820, and 591 (dynes ∙ sec/ cm^5^), respectively. This was negligible when compared with estimates in humans, with the chamber resistance equating to 0.65–5.86%, 1.23–6.86%, and 0.05–1.67% of the coronary resistance for the LAD, LCX, and RCA, respectively at varying flow rates and activity states. Therefore, the chamber served as a means to simulate the compliance of the distal coronary trees and to allow facile coupling with a set of known resistance catheters to simulate various physical activity levels.

**Conclusions:**

We have developed a method to create complex 3D printed patient specific coronary models derived from CCTA, which allow adjustable distal capillary bed resistances. This manufacturing approach permits comprehensive coronary model development which may be used for physiologically relevant flow simulations.

## Background

Three-dimensional printing (3DP) offers a unique opportunity to build geometrically accurate patient-specific vascular phantoms that can be used for device testing [1, 2], treatment planning [3], resident physician training [4] and physiological simulations [5, 6]. Recent literature has highlighted the advancements in additive and subtractive manufacturing that allow physicians to train and plan for procedures using patient specific phantoms [6–14]. Use of these phantoms to practice various approaches has shown promise as a method to improve clinical interventional outcomes and reduce the risk of periprocedural complications. Patient specific vascular phantoms fabricated via additive manufacturing can be used to visualize complex anatomies and create models for mock device deployment. Previous investigations [15–18] used phantoms from stiff photopolymers that lack compliance of arteries vital for device placement. To capture the flexible and compliant nature of the arteries, other investigators have followed similar approaches by fabricating a stiff 3D printed cast for silicone or polyurethane injection molding [19–24].

In the last decade, photopolymer 3DP has advanced with the addition of flexible photoresins, such as the Stratasys Tango family (Stratasys, Eden Prairie, MN) and the Visijet 3D Systems family (3D Systems, Rock Hill, SC). When combined with multi-material 3DP capability, this may allow development of vascular models where the mechanical properties of the anatomical structures might be replicated. A number of case studies have documented the feasibility of using 3DP vascular models where arterial wall mechanical properties such as compliance, stiffness, and hemodynamic pressure are preserved [20, 21, 25]. Another important application of the vascular phantoms are hemodynamics simulations where, in addition to the wall properties, special consideration must be paid to the boundary conditions, namely the inflow and outflow parameters. While the inflow may be controlled using programmable pumps, the outflow is dependent on the arterial network resistance. The distal arterial network presence is an important aspect for proper simulation of the distal resistance; however, representation of the arterioles and capillary bed using 3DP technology is not currently possible. This is due to both limitations in 3D imaging resolutions and 3DP capabilities. Inherent resistance and a compliance of the microvascular/ capillary bed are directly related to the hemodynamics in large and medium arteries, and therefore, simulation of these components in a 3D printed benchtop model design is crucial.

Regarding coronary 3DP models, the distal arterial resistance affects the flow rate through the coronary arteries and ultimately the pressure gradient along the diseased lesions and changes as an effect of activity state. The resistance is also specific to each of the three main coronary arteries and therefore they need to be treated as parallel systems. The coronary vascular bed consists of both coronary arterial resistance and coronary venous resistance which may be additive and positioned in series in a flow resistance circuit [26]. The compliance is controlled by both the elasticity and change in pressure within the arterial system and therefore regulation of this flow aspect is significant in hemodynamics simulation [18, 27, 28].

To address the distal resistance and compliance challenge, we have developed a method to create comprehensive 3D printed patient specific coronary models derived from coronary computed tomography angiography (CCTA) which maintain the majority of the large and medium sized coronaries. A model which includes the simulation of the distal resistance and the compliance of the coronary arteries within a benchtop system is beneficial in determining the effect these parameters have on the hemodynamics of the coronary arteries. Data collected from these optimized patient specific 3DP coronary models can be applied to validate fluid dynamic software, endovascular treatment planning, and other benchtop simulations [29]. To circumvent the imaging and 3DP limitations in the arteriole and capillary bed, we built a three-chamber outlet system in which each main coronary artery and its corresponding branches are collected to allow simulation of the arterial compliance and facile flow experiment control. The three-chamber design also allows decupling of the distal flow in each coronary tree and distal resistance adjustment via insertion of catheters of varying sizes distal to the compliance chamber. This enables simulation of capillary bed resistance for rest and hyperemic flow while recording pressure and flow rates through the main coronary arteries.

## Methods

**Fig. 1:**
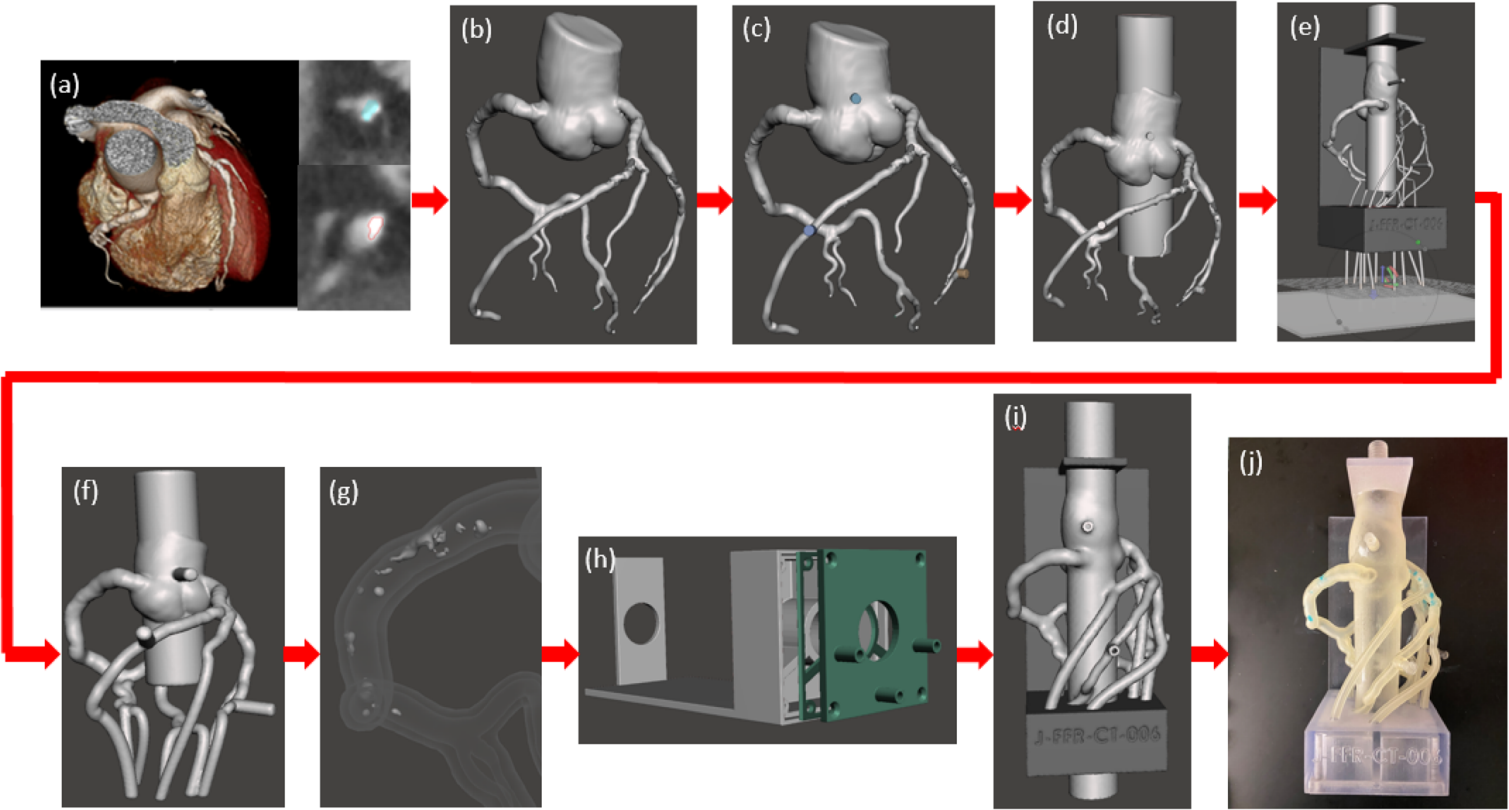
Model development process. **a** CCTA scans of the heart tissue and the three main coronary arteries were imported into Vital Images cardiac analysis application. The coronary arteries were segmented separately from the calcification using thresholding and contouring methods. **b** A stereolithographic [30] file was exported from Vital Images and imported into Autodesk Meshmixer and segmentation errors were removed. **c** Cylindrical meshes were appended to the aortic root and the diseased coronary artery for future pressure sensor connections. **d** The aortic root was extended at both the inlet and the outlet. **e** Vessel branches were extended through each of the three chambers and a plane cut was administered at the vessel outlets for parallel ends. **f** A 2 mm wall was generated and the lumen was hollowed out. **g** The calcification was solidified and subtracted from the vasculature. **h** A three-chamber support structure was imported into Autodesk Meshmixer. **i** Then model and support structure are aligned and ready to be printed. **j** The model is 3D printed, cleaned, and ready to be attached to a flow loop

**Fig. 2:**
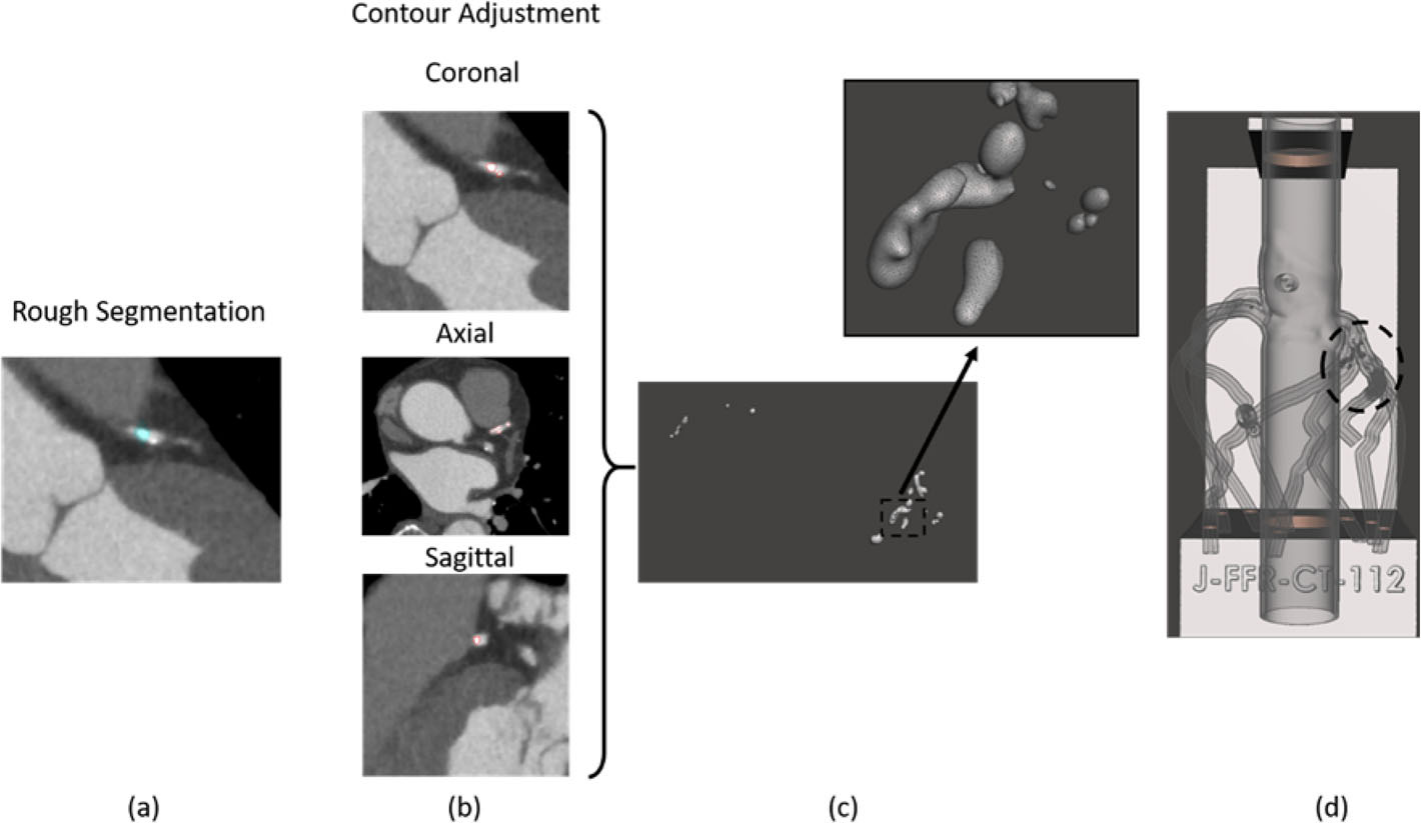
Calcification design. **a** The calcifications were segmented from Vitrea (Vital Images) separately from the rest of the coronary arteries using a rough segmentation. **b** Next, an expert user inspected and adjusted the contours in all three planes for every plaque. **c** Shows the overview of the plaques and enlarged details after the STLs geometries were imported into MeshMixer (**e**) Shows the top view of model. The dotted circle indicate the location of the plaques

The workflow we developed for this project is shown in Fig. 1. Five patients with coronary artery disease were used for this study from a data set of 75 acquisitions using the volumes with the least motion and blooming artifacts. Each patient underwent clinically indicated first generation 320-detector row CCTA (Aquilion ONE, Canon Medical Systems, Tustin, CA) with 0.5 mm slice thickness, automated tube current modulation, 100 kVp, and a reconstructed voxel size of 0.625 × 0.625 × 0.5 mm. Patient CCTA data was used within Vitrea (Vital Images, Minnetonka, MN) cardiac analysis application by expert users (fellows, surgeons, CT technicians) for automatic segmentation of the aortic root, Left Anterior Descending (LAD), Left Circumflex (LCX), and Right Coronary Artery (RCA) (Fig. 1a). The three main coronary arteries and branches were manually selected and included in the vascular segmentation using the select “artery tool”. The calcification was segmented separately from the vasculature through the “organ selection tool”, (Fig. 2a), and contrast thresholding was used to control inclusion of the entire plaque volume. To improve the accuracy of the calcification volume, we manually adjusted the segmentation contours in the CT slices, (Fig. 2b). The user spent between 20 min and 1 h on the anatomy segmentation, depending on the severity of the stenosis in the vasculature. Next, the vasculature and calcification segmented volumes were exported as separate stereolithographic files (STL) (Fig. 2c) [30].

The phantom will be 3D printed in a soft material to replicate vascular wall elasticity. To ensure anatomical position preservation and ease-of-use during experiments, a support structure was created in SolidWorks (SolidWorks Corporation, Waltham, MA) as previously described (Fig. 1h) [12]. The support is designed especially for the coronary tree and provides a 3 cm access hole for the aortic inflow and outflow, respectively. For flow management and distal resistance simulation implementation, we designed a three-chamber structure which collected the outflow of the main arteries and daughter branches. The chambers were sealed using a specially designed door and rubber gasket, which also allowed outflow of the collecting fluid. The design was implemented such that it would allow 3DP in one session. At this stage we also designed the aortic inflow and outflow as a cylinder (Fig. 1e). The cylinder is 2.6 cm in diameter to allow wall addition and is centered with the access hole in the support structure.

Next, we imported the STL files into Autodesk Meshmixer (San Rafael, California) for the removal of lumen segmentation artifacts, if present. The Robust Smoothing tool was used on rigid areas of the mesh where artifacts and errors occurred (Fig. 1b). Small cylinders were appended to the aortic root and each of the three main coronary arteries (Fig. 1c). These cylinders were later hollowed out for insertion of pressure sensors. The STL of the SolidWorks base was imported into Autodesk Meshmixer and manually aligned with the model. The alignment must be done using translation and rotation of the chamber only, not the artery. If the coronary tree is moved, then the alignment of the plaque with the lumen will be lost. Next, the inlet/outlet cylinder was separated from the support structure and combined with the aortic root using Boolean union, Fig. 1d.

One of the most challenging parts is to ensure that each main artery and branches end in the same chamber. For this, the end of each artery must be routed accordingly while minimizing the added length. A temporary flat disk was added distal to the coronary tree, parallel with the three-chamber back wall. The disk must be part of the same object as the coronary tree. The three main coronary arteries and branches were extended distally using the “add tube” function to append 1.2–1.8 mm diameter arterial extensions. By controlling the location of the tube on the added plane and the length, each artery can be routed through the desired chamber (Fig. 1e). Once this process is completed for each main artery and branches, the lumen, pressure ports and aortic inflow and outflow were extruded and to create a 2 mm wall, which preserved the coronary lumen and the geometry of the coronary arteries relative to the aortic root (Fig. 1f). A copy of the outer shell was created and subtracted from the support to allow perfect fitting of the model into the support structure and outflow orifices for the arteries and branches into the corresponding chambers. To create the arterial wall of the phantom, the inner lumen was selected, the surface normal was rotated 180 degrees and combined with the remaining outer shell. Outlets were created using the “cutting plane” function.

Next, we must ensure the calcification is properly inserted in the model. First, we increased the mesh density to avoid errors during 3D Boolean operations. The calcification meshing was increased using “make solid” function. A copy of the calcification was created, and the Boolean Difference Operation was performed between the vasculature and the calcification (Fig. 1g). This operation ensured there was no surface overlap between the arterial wall and the remaining calcification copy (Fig. 2d).

**Fig. 3:**
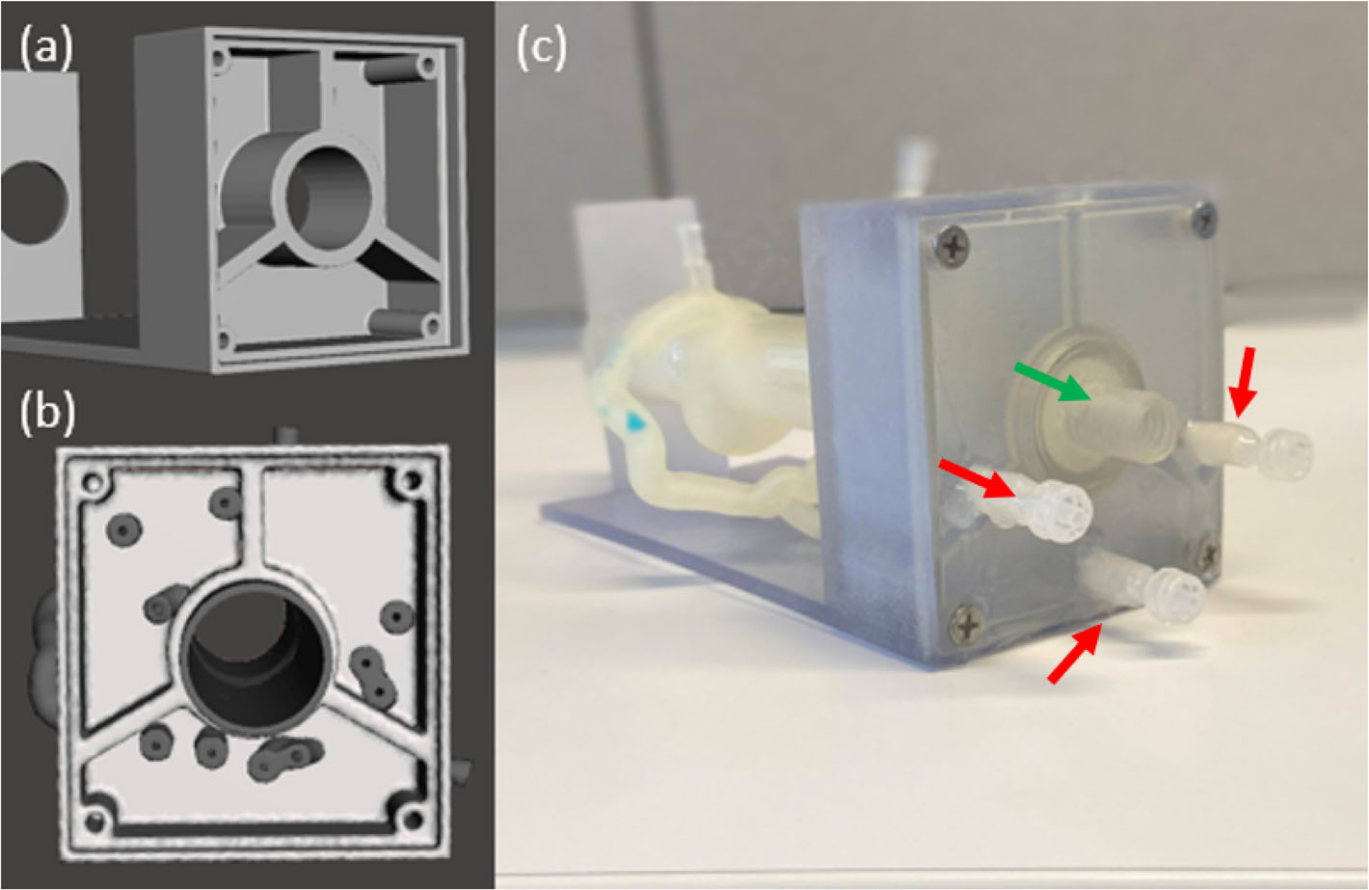
Three chamber design. **a** The three-chamber design was created in SolidWorks and then imported into Autodesk Meshmixer. **b** The vasculature was aligned with the three-chamber model in Autodesk Meshmixer. **c** The model was 3D printed and the chamber door was screwed on. Red arrows indicate luer connectors for catheters with known hydraulic resistance attachment. Green arrow indicates the connection to the ball valve for the programable pump

After mesh manipulation was completed (Fig. 1i), the model was 3D printed using the Stratasys Object 500 multi-material printer (Stratasys, Eden Prairie, MN). The vasculature was printed in Agilus, a soft elastic material within the arterial compliance range. The calcification and the support structure were printed in Vero, a hard material. The chamber door was printed in Vero with an Agilus rubber seal attached to it. The printed model was cleaned using the FORTI Support Removal system (PostProcess, Buffalo, NY), a process in which the model is submerged into an ultrasonic solution bath at a specific agitation level and time which varies based on the material being utilized. The models were also cleaned manually until all support is completely removed from the models. The models were heat dried and the aortic inlet and outlet connectors along with the pressure sensor connectors were adhered to the model. The aortic connectors were engineered to fit the inner diameter of the extended 3DP aorta, approximately 30 mm. The aortic valve was simulated at the inlet of the aorta using a 3DP ball valve inserted into the aortic inlet connector. Pressure sensor connectors were engineered to fit the 4 mm port created in the 3DP models with a luer outlet for the pressure sensor to tightly connect. A silicon glue was used to ensure a tight seal between the aorta and the inlet and outlet connectors as well as the pressure sensor connectors. The chamber door was then screwed into the chamber for a tight seal (Fig. 1j, Fig. 3). To prevent leakage during future benchtop flow testing, Agilus 3DP material was extracted from the cartridge using a 50 cc syringe in a low illuminated area and brushed onto all connectors. The model was then placed under a UV curing lamp to cure the added material layers. Since each time only a thin layer can be added, the process was repeated three times.

**Fig. 4:**
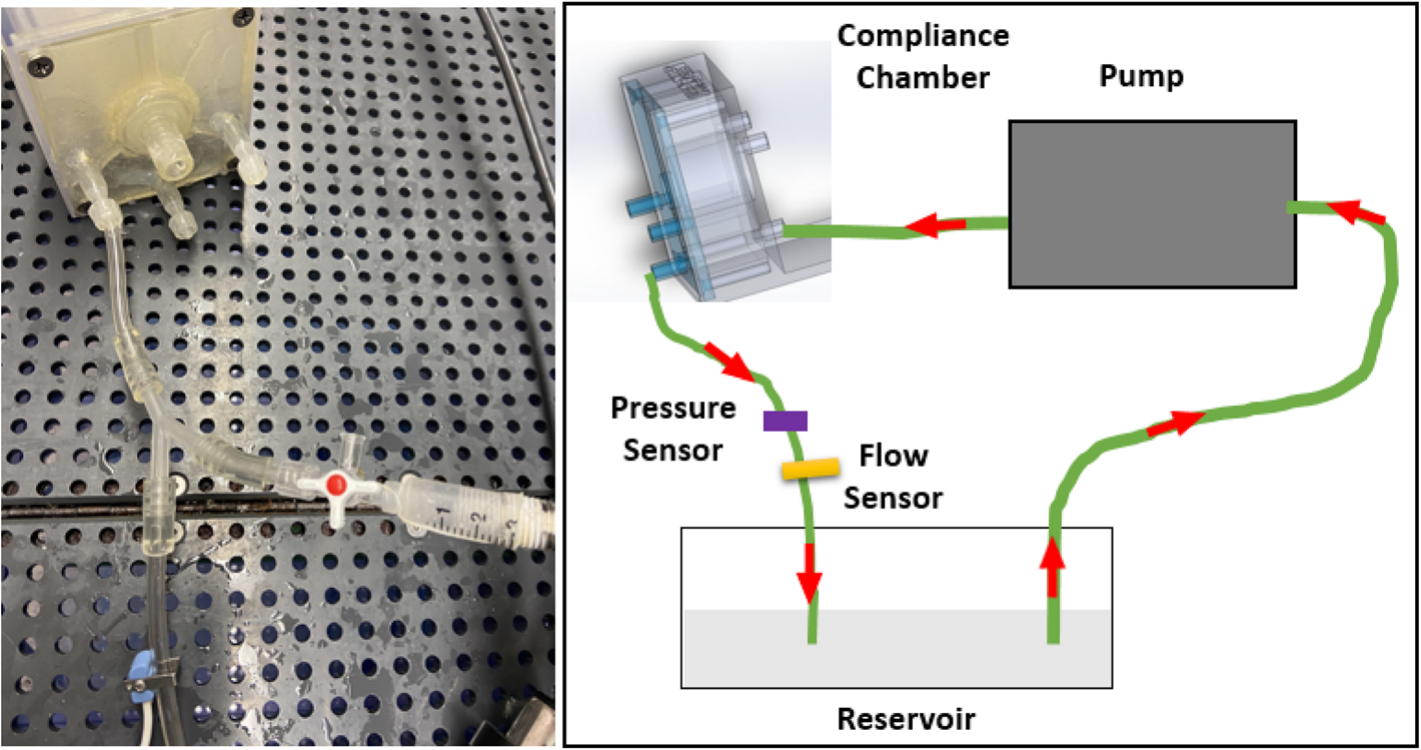
Stand-alone Chamber Resistance Testing Diagram. The intrinsic hydraulic resistance of each chamber was tested via a stand-alone 3D printed chamber without the coronary arteries and flow experiments were performed where constant flow was passed through each chamber section individually. An image of the benchtop setup as well as a systematic diagram of the flow setup is shown

To determine the intrinsic hydraulic resistance of each chamber, we created a stand-alone 3D printed chamber without the coronary arteries and performed a flow experiment where constant flow was passed through each chamber section individually. The 3D printed chamber template was connected to a constant flow loop using the CardioFlow 5000 MR programmable physiological flow pump (Shelley Medical Imaging Technologies, Toronto, Ontario, Canada), Fig. 4. A constant flow rate of 80–160 mL/min in increments of 20 mL/min was introduced into each of the three coronary chambers separately, and pressure differences and flow rates were recorded for each. Determination of the hydraulic resistance of each chamber is needed to determine the inherent effect that this would have on the flow-based experiments. The additional resistance they provide should be accounted for to avoid systematic errors.

**Fig. 5:**
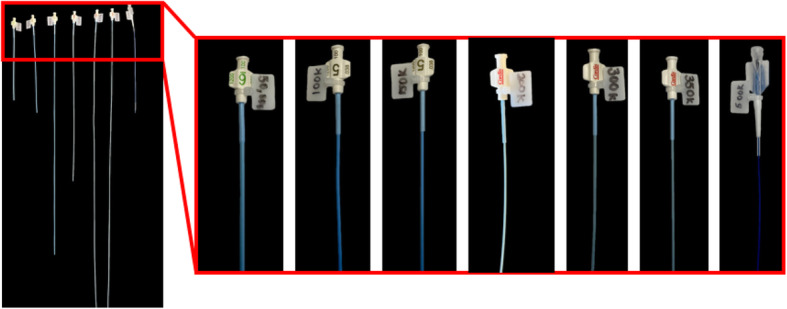
Distal Resistance Catheters. Resistances of 50,000–500,000 dynes ∙ sec/ cm^5^ were obtained within catheters that were cut to a specific length and specific diameter chosen. They were then connected to the distal end of the three chamber design simulating coronary distal resistance

Catheters of varying diameters and lengths were connected distal to the support chamber to match the lumped parameter models of the coronary vascular beds for simulations of the thoracic aorta with the coronary arteries at rest, during light exercise, and during moderate exercise [26]. The distal hydraulic resistance of each of the three main coronary arteries is inversely proportional with the level of physical activity. These varying degrees of activity induce changes in coronary dilation to increase coronary flow, and therefore, the differing resistances occur. As the physical activity level increases, the resistance decreases to provide higher coronary flow through the coronary arteries. These resistance levels, as well as the compliance, have been computed by Kim et al. [27] and they are summarized in Table 1. To simulate and allow quick adjustment of the distal flow conditions in the phantoms, we implemented use of calibrated catheters, where various resistance levels were achieved by changing the catheter size and the length based on the calculations in Table 1. Where the majority of the catheter lengths for 4, 5, and 6 French catheters are feasible, a few catheter lengths are greater than the catheter length options commercially available. In this case, a smaller French size catheter would be needed to replicate the specific average resistance, shown with an asterisk (*) in Table 1. These catheters are designed for quick exchange using the luer ports indicated by red arrows in Fig. 3. Since the values in the Table 1 are focused on a relatively small sample, we created a set of catheters with resistances in multiples of 10,000 dynes*sec/cm^5^. We confirmed the values experimentally, while measuring the flow and pressure drop in the same manner as the described above for the 3D printed chamber, the catheter final length was adjusted to ensure that the hydraulic resistance was achieved to the nearest 10,000 dynes*sec/cm^5^. Resistances between 50,000 and 300,000 dynes ∙ sec/ cm^5^ were created in 4, 5 and 6 French catheters at 17–112 cm lengths (Fig. 5).

**Table 1:** Catheter radii, and length to match cardiac coronary resistance at various levels of exercise

Condition	Vessel (cm^**3**^/sec)	Average Resistance [26] (dynes*sec/cm5)	Catheter Radii (cm)	Catheter Length (cm)
**Rest**	LAD	254,250	0.06	34.9
			0.071	68.5
			0.09	176.95^a^
	LCX	137,000	0.06	18.8
			0.071	36.9
			0.09	95.3
	RCA	479,000	0.06	65.8
			0.071	129.1^a^
			0.09	333.3^a^
**Light Exercise**	LAD	71,250	0.06	9.7
			0.071	19.2
			0.09	49.5
	LCX	81,750	0.06	11.2
			0.071	22.0
			0.09	56.8
	RCA	121,000	0.06	16.6
			0.071	32.6
			0.09	84.2
**Moderate Exercise**	LAD	29,000	0.06	3.9
			0.071	7.8
			0.09	20.1
	LCX	29,300	0.06	4.0
			0.071	7.8
			0.09	20.3
	RCA	50,300	0.06	6.9
			0.071	13.5
			0.09	35.0

^a^Indicates that a catheter of that length is not typically engineered and commercially available and smaller Fr size must be used to replicate that specific average resistance

## Results

We created five 3D printed patient specific models for this research, including the three chambered support design. The estimated mean times to build each 3D printed multi-material model were as follows: 30 min for segmentation, 1.5 h for model design, 60 min for chamber design, 24 h to print one tray of five models.

**Fig. 6:**
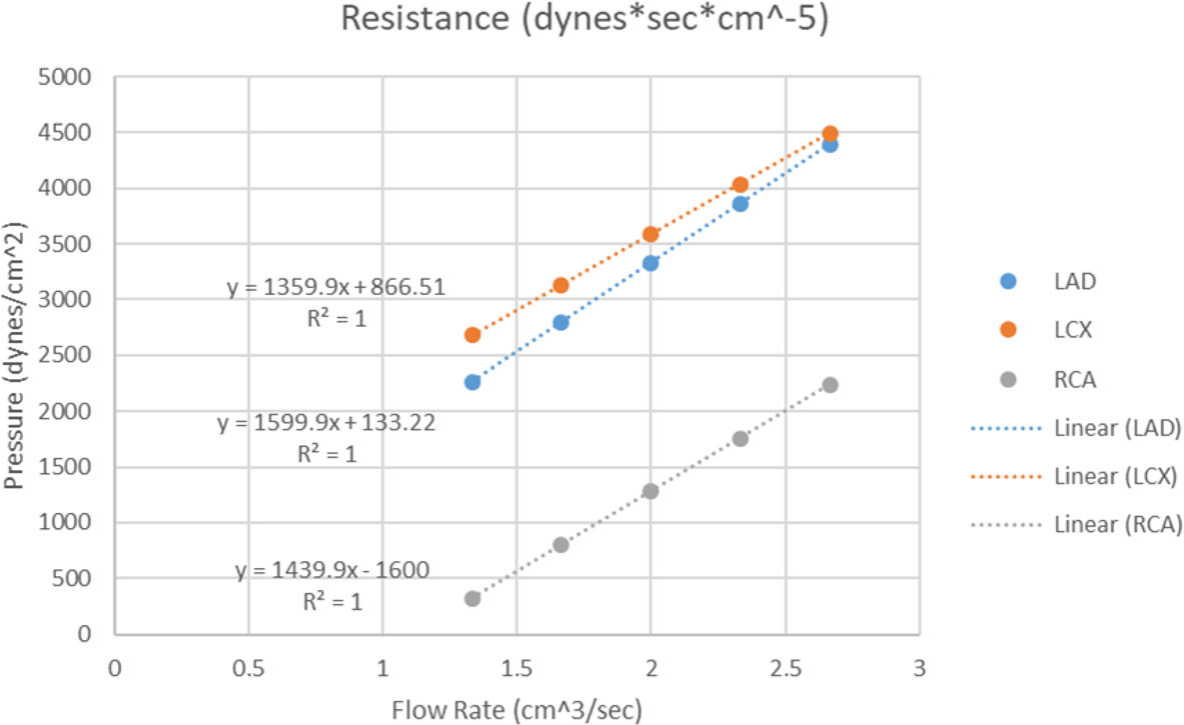
Three chamber resistance. The resistance of the chamber was determined by recording the pressure through a tube at flow rates from 1.33–2.66 cm^3^/sec within the LAD, LCX and RCA chambers

Typical pressure wave measurements at flow rates between 80 and 160 mL/min for each main coronary artery were recorded. The resistance of the chamber alone without any coronary arteries connected was determined based on the input flow rate and the pressure difference to measure the impact it would have on the benchtop system once connected to our 3DP patient specific phantom flow loop (Fig. 6). There was one inlet and one outlet per chamber in a mockup chamber where no arteries were connected. The mean resistance of the chamber for the LAD, LCX, and RCA, were 1671, 1820, and 591 dynes*sec/cm^5^, respectively. The mean resistances of the LAD, LCX, and RCA themselves within a body at rest are 250,000, 150,000, and 500,000 dynes ∙ sec/ cm^5^, respectively [26]. Table 2 shows the percentage of the resistance of the chamber for each of the three chambers corresponding to the LAD, LCX, and RCA compared to the resistance of each individual coronary artery at physiologically relevant flow rates of 1.33–2.67 cm^3^/sec. The predicted results obtained should be linear as we were pumping a constant flow through the chamber, we obtained the pressure, and then calculated the resistance for each flow rate.

**Table 2:** Chamber Resistance as a Percentage of the Coronary Resistance

Coronary Artery	Flow Rate (cm^**3**^/sec)	R (%)	E1 (%)	E2 (%)
LAD	1.33	0.66	2.38	5.86
LAD	1.66	0.66	2.35	5.79
LAD	2.00	0.65	2.33	5.74
LAD	2.33	0.65	2.32	5.71
LAD	2.66	0.64	2.31	5.68
LCX	1.33	1.46	2.45	6.85
LCX	1.66	1.37	2.29	6.41
LCX	2.00	1.30	2.19	6.11
LCX	2.33	1.26	2.11	5.90
LCX	2.66	1.22	2.06	5.75
RCA	1.33	0.05	0.19	0.47
RCA	1.66	0.10	0.39	0.95
RCA	2.00	0.13	0.52	1.27
RCA	2.33	0.15	0.62	1.49
RCA	2.66	0.17	0.69	1.66

Data is presented as the resistance of the chamber as a percentage of the resistance of the coronary artery during which the coronary vascular bed is at rest (R), during light exercise (E1), and during moderate exercise (E2). LAD indicates the Left Anterior Descending, LCX indicates the Left Circumflex and RCA indicates the Right Coronary Artery

## Discussion

This paper describes the development of a method to create comprehensive 3D printed patient specific coronary models derived from CCTA. These models give us the ability to replicate the geometry of arteries with submillimeter accuracies while providing means to control distal flow conditions [29]. Using the proposed three chamber outlet system, we can control separately the distal resistance of each coronary tree subsection and adjust within human relevant ranges. The addition of the catheters of varying resistances allowed for simulation of the capillary beds that is not currently possible using 3D printing technology. The resistance of the chamber proved to be negligible, with the chamber resistance equating to 0.65–5.86%, 1.23–6.86%, and 0.05–1.67% of the coronary resistance for the LAD, LCX, and RCA, respectively, at flow rates within typical rest to moderate physical activity. It can be concluded based on these results that the chamber did not add significant distal resistances to the three individual coronary arteries, while serving its purpose as a compliance chamber to each of the LAD, LCX, and RCA trees. This deems importance as catheters of varying sizes were inserted into the benchtop set up to function as structures simulating the specific distal resistance of each coronary artery.

3DP offers a unique opportunity to build flexible vascular models with patient specificity for device testing, treatment planning, clinical resident training and physiological simulations. By optimizing these models both geometrically and hemodynamically, much promise is shown for this tool to improve clinical interventional outcomes as well as allow for a better understanding of coronary artery disease flow complexity. From the CCTA scan to being 3D printed, geometric manipulation is kept at a minimum to allow for highly accurate patient specific models. These phantoms are created to be put into a system that is highly interchangeable in regards to flow rates and pressures and can withstand pressures between 80 and 140 mmHg. To circumvent the inability to image and 3D print fine capillary structures, we developed a system where the effect of such structure on the flow is simulated using adjustable distal resistance. Our system incorporates the distal arterial compliance through the integration of the three chambered structure in which we are able to regulate the change in pressure as a function of volume, determined individually for each of the three main coronary artery’s capillary beds. The distal resistance is simulated through the connection of catheters to the chamber (Fig. 3c) of varying diameters and lengths to account for the specific resistances of the LAD, LCX, and RCA at changing activity states.

There are a few limitations related to the phantom design process. Regarding imaging, the CT imaging accuracy was reduced by the voxel size with the 3DP resolution of 200 μm surpassing the spatial resolution of the CCTA of 630 μm. A limitation of this study is using five CCTA volumes with reduced artifacts from a set of 75. Since most of the coronary arteries are between 4- and 5-mm diameter, a small error in the segmentation can result in significant changes in flow. Thus, only images with reduced motion artifact and calcium burden should be used or advanced imaging techniques which will allow alleviate these limitations. Involving the segmentation process, there may have been some errors in exclusion of hard-to-detect, soft plaque when segmenting the calcification from the vasculature, thus altering the diameter. We cross-validated this process between two users to avoid significant errors [31]. In addition, in the process of small branch and segmentation artifact elimination, we sculpted the mesh within Autodesk Meshmixer manipulating single triangular vertices, which might have created minor geometric alteration. During the process of inlet and outlet extension of the aortic root, we tried to align the cylinder with the mesh so that the outer ridge of the ostium was not affected to allow for normal turbulent flow within the aorta, but minor error possibly occurred during cylinder alignment. Additionally, as most of the distal ends of the arteries are in close proximity to the chamber structure, but not well separated by specific chamber, we added tubing to extend the coronary artery branches to allow optimal separation into the corresponding chamber (e.g. LAD, LCX, RCA). The added tube consisted of one centimeter or less of distal extension. The added segment had one millimeter larger diameter, than the distal tip of the artery to reduce the overall contribution of resistance. Another potential limiting factor is that the elastic material in which the phantoms are printed allows deformations, which may affect the accuracy of benchtop testing done on the models over time.

Other limitations are related to the manipulation of the distal resistance of the coronary arteries. Though the resistance of the three chambered structure proves to be 0.050–6.859% of the coronary resistance, it still adds to the total resistance of the phantom. Therefore, to account for this added resistance, the user should subtract the chamber resistance from the coronary resistance in each of the three main coronary arteries to give the resistance needed distally from the catheters. Also, the catheters used in our models rounded the resistance values recorded from Kim et al. [26], inducing an error of ~ 2%.

## Conclusions

We have developed a method to create comprehensive 3D printed patient specific coronary models derived from CCTA to replicate the hemodynamics in the arteries. With these models, we have the capability of controlling distal flow conditions using a three-chamber outlet system in which each main coronary artery and its corresponding branches are adjusted individually. Our results present an innovative method in which geometric modeling as well as physiologically accurate blood flow conditions are able to be met in this coronary vascular modelling system. Through the use of 3DP, we were able to create patient specific coronary benchtop models with geometrically accurate coronary arteries with the addition of a compliance chamber and distal coronary artery resistance components simulating the capillary beds.

## Data Availability

The datasets used and/or analyzed during the current study are available from the corresponding author on reasonable request.

## Abbreviations

CCTA: Coronary Computed Tomography Angiography
LAD: Left Anterior Descending
LCX: Left Circumflex
RCA: Right Coronary Artery
STL: Stereolithographic
MIVI: Minimally Invasive Vascular Intervention
